# Dimensional Accuracy of Dental Models for Three-Unit Prostheses Fabricated by Various 3D Printing Technologies

**DOI:** 10.3390/ma14061550

**Published:** 2021-03-22

**Authors:** Soo-Yeon Yoo, Seong-Kyun Kim, Seong-Joo Heo, Jai-Young Koak, Joung-Gyu Kim

**Affiliations:** 1 Department of Prosthodontics and Dental Research Institute, Seoul National University Dental Hospital, School of Dentistry, Seoul National University, 101 Daehak-ro, Jongno-gu, Seoul 03080, Korea; sy0502@snu.ac.kr (S.-Y.Y.); 0504heo@snu.ac.kr (S.-J.H.); young21c@snu.ac.kr (J.-Y.K.); 2 Sense Dental Laboratory, 1104, Seoul Soop IT-Valley, 77, Seongsuil-ro, Seongdong-gu, Seoul 04790, Korea; senselab@naver.com

**Keywords:** 3D printing, digital light processing (DLP), multi-jet printing (MJP), stereo-lithography apparatus (SLA), dimensional accuracy

## Abstract

Previous studies on accuracy of three-dimensional (3D) printed model focused on full arch measurements at few points. The aim of this study was to examine the dimensional accuracy of 3D-printed models which were teeth-prepped for three-unit fixed prostheses, especially at margin and proximal contact areas. The prepped dental model was scanned with a desktop scanner. Using this reference file, test models were fabricated by digital light processing (DLP), Multi-Jet printing (MJP), and stereo-lithography apparatus (SLA) techniques. We calculated the accuracy (trueness and precision) of 3D-printed models on 3D planes, and deviations of each measured points at buccolingual and mesiodistal planes. We also analyzed the surface roughness of resin printed models. For overall 3D analysis, MJP showed significantly higher accuracy (trueness) than DLP and SLA techniques; however, there was not any statistically significant difference on precision. For deviations on margins of molar tooth and distance to proximal contact, MJP showed significantly accurate results; however, for a premolar tooth, there was no significant difference between the groups. 3D color maps of printed models showed contraction buccolingually, and surface roughness of the models fabricated by MJP technique was observed as the lowest. The accuracy of the 3D-printed resin models by DLP, MJP, and SLA techniques showed a clinically acceptable range to use as a working model for manufacturing dental prostheses

## 1. Introduction

Digital models can be used to manufacture various dental appliances including fixed prostheses [1]. The impressions made by digital oral scanners enable the three-dimensional (3D) modelling of teeth by a computer and the fabrication of fixed prostheses without conventional working models. It is understood that the fit of fixed prostheses is the most important requirement for its stability and good prognosis. However, this recent development meant that the fit between the abutment and fixed prostheses cannot be determined until a clinician places a restoration in the oral cavity of a patient because it is manufactured only as a digital computer model. Therefore, fabrication of real working models by digital files can be recommended for verifying and correcting ideal fit before delivery. 

There are two kinds of new ways to manufacture a dental working model with a scanned digital file; the first is a model fabricated by computer numerical control (CNC) milling machines and the other is by a 3D-printing technique. The previous study showed that the 3D printer could fabricate concave and intricate geometry that is often not achievable by milling [2]. Thus, if resin models are manufactured with oral scanned files by the 3D-printing technique to enhance fit and accuracy of fixed prostheses, the fit of fixed prostheses can be adjusted and confirmed before being delivered. In other words, 3D-printed resin models can be good alternatives to conventional working models in the dental laboratory process. 

There are a number of 3D printing technologies—fused filament fabrication (FFF), selective laser sintering (SLS), stereo-lithography apparatus (SLA), digital light processing (DLP), Multi-Jet printing (MJP) technique, and so on. FFF technology was the most common due to relatively inexpensive costs, although the surface was less accurate and blurred rough [3,4]. The SLS method provides design freedom, but the surface created by SLS is rough and material options are limited. SLA and DLP both work with the polymerization of photosensitive resin from the bottom of a tank [1]. The DLP technology works with a plate projector which polymerizes an entire layer, whereas SLA uses a single point laser to polymerize [5]. After the expiry of the patent of SLA technology held by 3D systems (Rock Hill, SC, USA), SLA became one of the most common fabrication methods due to the high resolution, accuracy, clear detail, and smooth surface finish it could produce. DLP techniques have also been extensively used in all kinds of industrial fields. MJP, also known as Multijet (MJ) or PolyJet (PJ), jets photopolymer droplets, and UV light subsequently solidifies the polymer to form a 3D model. MJP is known to be more precise than other 3D-printing techniques, but it is more time consuming and expensive than other fabrication methods [6]. 

The term ‘accuracy’ in 3D printing is used when both trueness and precision are achieved, according to ISO 5725-1:1994/Cor 1:1998. The trueness of a measurement method is mentioned when it is possible to conceive of a true value for the property. The need to consider precision arises because tests performed on presumably identical materials under presumably identical circumstances do not, in general, lead to identical results. This is attributed to unavoidable random errors inherent in every measurement procedure. In short, trueness in this study refers to the closest results of the 3D-printed models with the reference model, whereas the precision refers to the closest results under the different replicas by one printing technology [3]. The main outcome for printing accuracy can be shown as the root mean square (RMS) value which is defined as the square root of the mean square (arithmetic mean of the squares of a group of values) between two areas.

The accuracy of a 3D-printed model can be affected by the total errors that occur throughout the overall fabrication process; the model scanning imaging, image segmentation, standard tessellation language (STL) file transition, STL post-processing, slicing of the STL file for 3D printing, 3D printing itself, and post-processing. All these steps are severely dependent on the software, the 3D printer, and after all, the user [7,8,9,10,11]. To reduce errors, digital workflow needed to be simplified, and that was what was done in this present study. 

In a previous study that compared the accuracy of intraoral and desktop model scanners, the intraoral scanning method exhibited twice as many 50-μm deviations as the desktop scan method. This may be due to intraoral humidity, patient movement, and limited intraoral spaces [12]. According to Son’s study, desktop scanners showed more accurate scanned data than intraoral scanners [13]. Therefore, by using desktop scanner for scanning and producing the STL image file, the comparison of the 3D-printed model itself can be focused on, fabricated by different technologies. 

The manual measurements of 3D-printed models are also influenced by the variability of the operator, and there is difficulty in repeatedly selecting the exact landmarks [14]. Although no measurement technique is error-free, computer-aided measurement methods can be beneficial in this case. Through superimposition in computer-aided program with scanned files of 3D-printed resin models and the reference file, errors of measurement can be reduced and the data produced by 3D-printed models can be focused on.

Few studies focused on the 3D-printed models used for fabrication of dental prostheses, such as fixed partial dentures and inlays. These models require higher accuracy than those used for diagnosis or orthodontic uses because the criteria of clinically acceptable marginal discrepancy of fixed prosthesis is only 120 μm [15,16]. Therefore, further studies on the accuracy of digitally-produced models are required. In this study, the aim was to evaluate the differences on the accuracy of 3D-printed models produced by the DLP, MJP, and SLA techniques—which are used for three-unit fixed prosthesis—by comparing RMS values of trueness and precision as well as analyzing marginal deviations and distance to proximal contact on two-dimensional (2D) planes.

## 2. Materials and Methods

### 2.1. The Preparation of 3D-Printed Models

**Figure 1:**
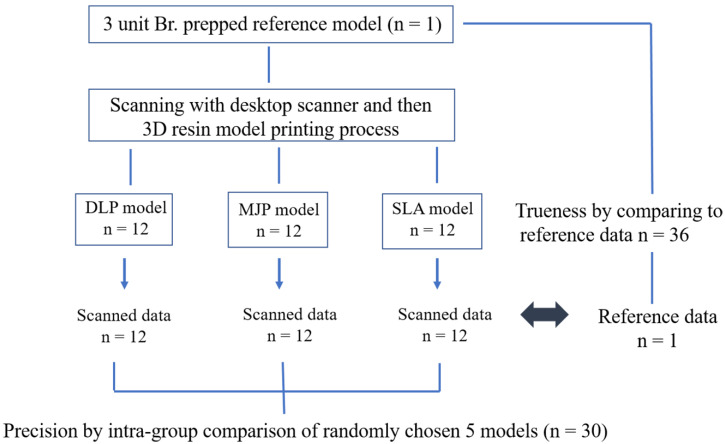
Flow chart of this study. 3D, three-dimensional; DLP, digital light processing; MJP, Multi-Jet printing; SLA, stereo-lithography apparatus.

**Figure 2:**
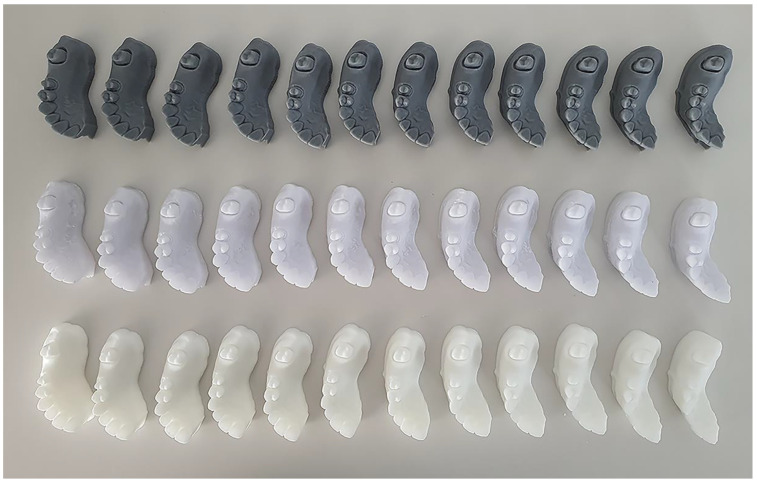
3D-printed resin models (*n* = 36) of this study. First row; DLP models, second row; MJP models; third row; SLA models.

In this study, we prepped of molar (#27) with long chamfer margin and premolar (#25) with deep chamfer margin for 3-unit fixed prostheses. This reference model was scanned by industrial 3D scanner (E4 lab scanner, 3Shape, Copenhagen, Denmark) with a resolution of Blue LED and 2 × 5 Mpx, thus, the reference STL file was obtained. The industrial 3D scanner from the manufacturer validated an accuracy of 7 µm according to ISO 12836 [17]. The digital models were replicated 12 times (*n* = 36) using the reference STL file by each of the DLP, MJP, and SLA techniques shown in Figure 1 and Figure 2. The parameters set for the 3D printers in each group are shown in Table 1. Thirty-six 3D-printed models were also scanned with industrial desktop 3D scanner. In previous studies, the surfaces of the DLP, MJP, and SLA casts were lightly dusted with powder to reduce light reflection; however, in the present study, for accuracy of measurements, we used a desktop scanner which does not need a scan spray.

### 2.2. The Dimensional Accuracy Measurements of 3D-Printed Models

**Figure 3:**
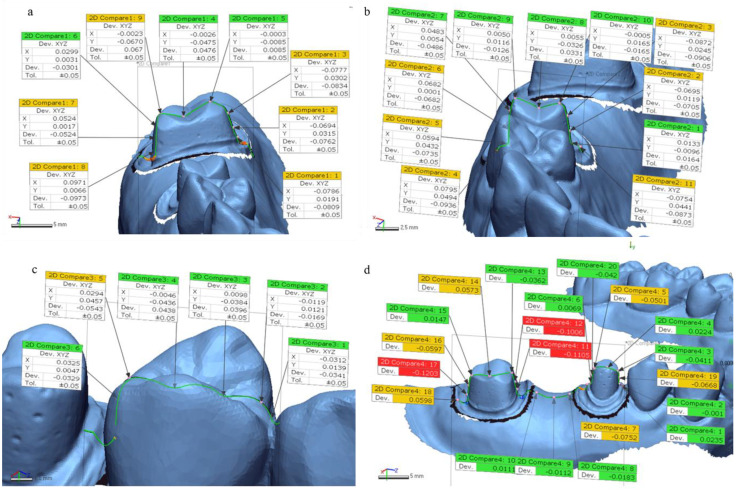
Four landmarks for measurement of trueness on 3D-printed models with the reference file. (**a**) molar tooth at buccolingual section. (**b**) Premolar tooth at buccolingual section. (**c**) Proximal contact to approximate tooth. (**d**) Molar and premolar teeth at mesiodistal section.

3D-printed models were analyzed for the accuracy at 4 landmark positions and assessed deviations on margins of prepped teeth and proximal contact area, shown in Figure 3. To measure the accuracy (trueness; inter-printer and precision; intra-printer reliability) of abutment teeth and distances to approximate teeth, the datasets were superimposed through a best-fit alignment method using a 3D inspection software of Geomagic (release 2018, Geomagic control X, 3D Systems, Rock Hill, SC, USA), according to recommendation of ISO-12836 [17]. Since a variety of 3D inspection software are aligned using different protocols, 3D analysis results may differ. 

Trueness was determined by comparing the measurements on 3D-printed models to those on the original image file (STL file of reference model) (*n* = 36). The precision of the 3D-printed models was evaluated on 5 randomly chosen samples by superimposing scan data within each group (*n* = 30) (Figure 1). The quantitative values were automatically calculated by the 3D analysis program based on RMS. The RMS values were used to verify the mean of the positive and negative values using the following formula:

$$\frac{1}{\sqrt{n}}\times \surd \sum_{i=1}^{n}(Xief-Xi)2$$

where *n* is the sum of the measured points, Xief is the measurement point of *i* of the reference model, and *Xi* is the measurement point of i of the dataset of the 3D-printed test models. 

**Figure 4:**
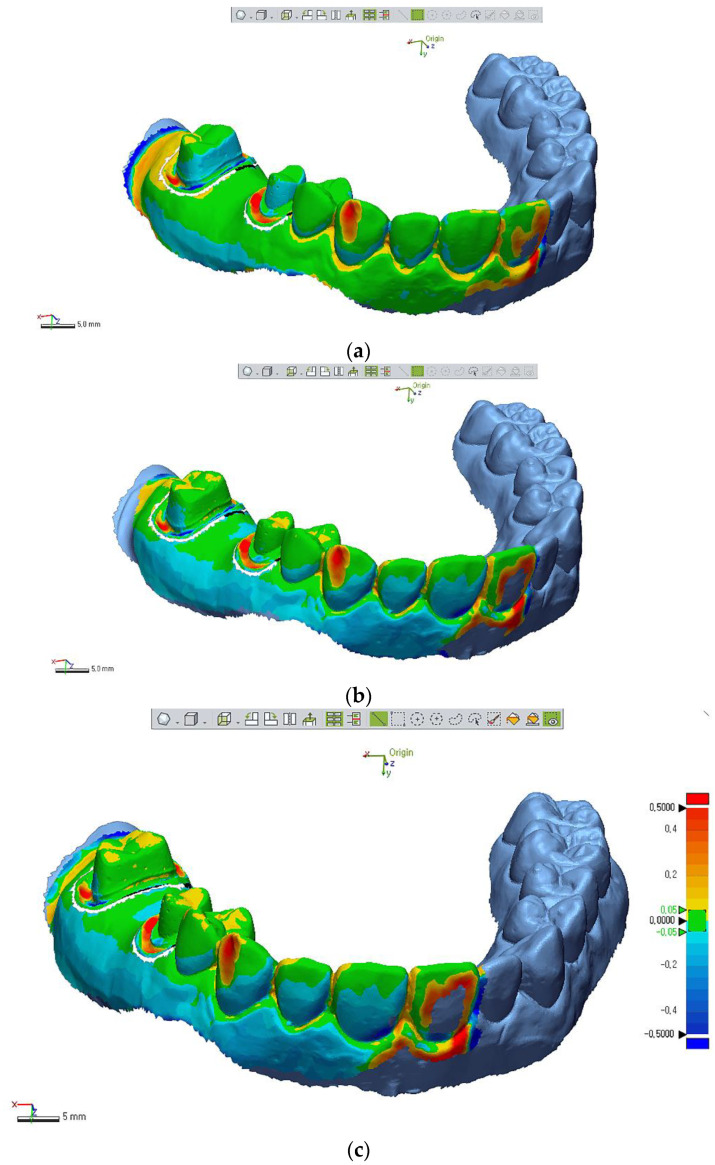
Color maps of 3D model superimpositions of each printed test model STL file with the original reference file. Table 50. μm expressing green colors, and max/min critical range at ±500 μm representing dark red or blue colors. The Scheme 0. to 0.5 mm. (**a**) DLP cast. (**b**) MJP cast. (**c**) SLA cast.

We set the tolerance as ±50 µm according to previous studies [18,19]. The percent of points within the tolerance level (inTOL; nominal ±50 µm) were also calculated with respect to accuracy for printed models. A color map expressing visual deviation was set with 20 color segments. The color map showed the amount of deviations between the test model STL file and the reference file [20]. The range of the maximum and minimum nominal values was set at 50 µm, and the range of the maximum and minimum critical values was set at 500 µm (Figure 4).

**Figure 5:**
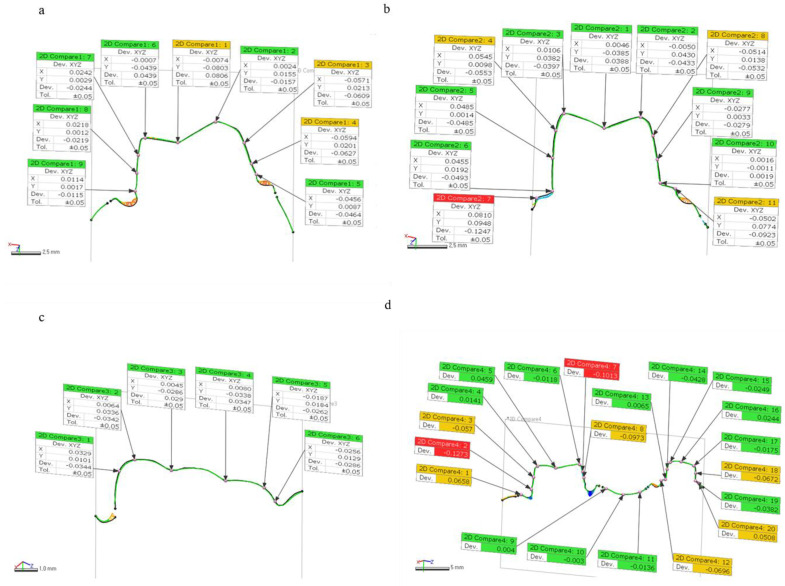
Measurements of deviations in two-dimensional (2D) planes. (**a**) Marginal deviations of molar teeth at a buccolingual plane. (**b**) Marginal deviations of premolar teeth at a buccolingual plane. (**c**) Deviations at proximal contact to approximate tooth. (**d**) Marginal deviations of molar and premolar teeth at a mesiodistal plane.

After best fit alignments of the test STL file with reference STL file, we sliced 3D model images to 2D planes (buccolingual and mesiodistal planes). We calculated deviations through Geomagic software at 4 measurement positions: margin of prepped molar and premolar teeth (buccolingual and mesiodistal planes), and proximal contact to an approximate tooth (Figure 5). To assess the deviations at margins and proximal contact of 3D-printed models, the absolute deviation values of 2 marginal points in prepped molar, 4 marginal points of prepped premolar, and 1 point of proximal contact, were analyzed.

### 2.3. The Measurements of Surface Roughness on 3D-Printed Models

We measured roughness of 3D-printed models by DLP, MJP, SLA techniques using confocal laser scan microscope (LSM800, ZEISS, 07745 Jena, Germany) because surface roughness could affect the differences of accuracy. 

### 2.4. Statistical Analyses

Statistical analyses were conducted using SPSS (IBM Statistics, v.25.0; IBM Corp, Armonk, NY, USA), and significance level was set at 0.05. Normal distribution of all data was examined through the Shapiro–Wilk test and homogeneity of variance with Levene’s test. As a result, the deviation values in 2D planes and inTOL (%) for precision between the 3D printer groups were analyzed using one-way ANOVA and Tukey HSD test as post hoc (significance level at *p* < 0.05), while differences of RMS values and inTOL (%) for trueness at 4 landmark 3D areas between printers were analyzed using Kruskal–Wallis H test. The Mann–Whitney U-test and Bonferroni correction were used for post-testing (*p* < 0.05/3 = 0.017).

The Intraclass Correlation Coefficient (ICC) value is the reliability calculated by the raters’ measurements. The ICC means reproducibility if the test is repeated several times [21]. The ICC value was also used to show the level of precision, at 95% confidence interval in this study.

## 3. Results

### 3.1. Trueness of 3D-printed Models

We analyzed accuracy (trueness and precision) through RMS values that are automatically calculated through 3D inspection software at 4 measurement positions (Figure 3). As shown in Table 2, for overall 3D analysis at molar position, there were significant differences in mean RMS values of trueness among all 3 techniques: DLP (0.117 ± 0.007 mm), MJP (0.096 ± 0.029 mm), SLA (0.101 ± 0.004 mm) (*p* < 0.001). For overall 3D analysis at premolar position, MJP (0.054 ± 0.002 mm) technique showed the most accurate result in comparison with DLP (0.064 ± 0.009 mm) and SLA (0.066 ± 0.005 mm) techniques (*p* < 0.001). For overall 3D analysis at proximal contact area, DLP (0.052 ± 0.011 mm) and MJP (0.059 ± 0.012 mm) techniques showed more accurate results than SLA (0.078 ± 0.021 mm) technique (*p* < 0.001). For overall 3D analysis at mesiodistal section (molar to premolar), DLP (0.100 ± 0.022 mm) and MJP (0.098 ± 0.010 mm) techniques showed better results compared to SLA (0.123 ± 0.008 mm) technique (*p* < 0.001). 

The inTOL (%) of trueness for 3D-printed models showed significant differences between printing techniques as shown in Table 3 (*p* < 0.05). MJP technique (88.166 ± 5.185%) showed the highest percent within the tolerance 0.05 mm level. Consequently, the 3 different 3D printing techniques in present study showed significant differences in trueness of RMS measurements and inTOL (%). 

### 3.2. Deviations of 3D-Printed Models at 2D Planes

We examined the absolute values of deviations, at margins of prepped molar and premolar area, and to proximal contact at 2D planes (Figure 5). For marginal deviations at a buccolingual plane of prepped molar, DLP (0.100 ± 0.022 mm), MJP (0.0291 ± 0.020 mm), SLA (0.070 ± 0.015 mm) groups showed significant differences (*p* < 0.001). For marginal deviations at mesiodistal plane of prepped molar, DLP (0.109 ± 0.015 mm), MJP (0.020 ± 0.012 mm), SLA (0.051 ± 0.044 mm) groups also showed significant differences (*p* < 0.001). However, Table 4 showed marginal deviations of prepped premolar had no significant difference in both buccolingual and mesiodistal planes (*p* = 0.625, 0.996 respectively). For deviations at proximal contact area on mesiodistal plane, MJP (0.009 ± 0.009 mm) technique showed the lowest value compared to DLP (0.036 ± 0.015 mm) and SLA (0.036 ± 0.015 mm) groups (*p* < 0.001). 

Considering the deviation values ((+); expansion, (−); contraction) at each point and 3D color map, the all 3D-printed casts showed contraction buccolingually, and MJP casts showed least change. In posterior regions (molar area), contraction was more than anterior regions (premolar area). 

### 3.3. Precision of 3D-Printed Models

Table 5 showed the DLP (0.244 ± 0.117 mm), MJP (0.272 ± 0.103 mm), and SLA (0.249 ± 0.069 mm) techniques exhibited no significant differences on RMS values of precision (*p =* 0.381). Table 6 showed the inTOL (%) of precision for 3D-printed models also showed no statistical differences between printing techniques (*p* = 0.643). Precision of the 3D-printed models was also measured using ICC. The ICC of the three test groups were 0.996 (DLP), 0.955 (MJP), and 0.992 (SLA) as shown in Table 7. All test groups exhibited an excellent level of precision, based on 95% confident interval of the ICC estimation.

### 3.4. Surface Roughness of 3D-Printed Models

Table 8 showed the mean surface roughness of 3D-printed models by MJP technique (Ra: 2.345 ± 0.374 µm), which observed the smoothest compared to the DLP (Ra: 4.123 ± 2.531 µm) or SLA (Ra: 7.75 ± 2.478 µm) technique. 

## 4. Discussion

In the analysis process of this study, the analysis software showed 3D deviation data that consisted of automatically calculated RMS, average (+) and average (−) values on 4 measurement sections, and individual deviation values ((+); expansion or (−); contraction) at multiple measurement points of 2D planes (Figure 3 and Figure 5). In this study, only RMS values were used for examining 3D deviations of printed models and absolute deviational values at 2D planes (buccolingual and mesiodistal) because in quantitative evaluation, if the average (+) and average (−) deviations express an equal distribution, the sum values will be close to zero, which make results confusing. In addition to that, the color maps for a qualitative inspection were segmented with 20 colors, showing contraction or expansion at best fit alignment of test STL files with the reference STL file. 

Hazeveld et al. concluded that measurement differences of less than 250 µm were clinically acceptable values since the tolerances for manual measurements were almost identical to that value [22]. This study calculated the accuracy of printed models through superimpositions of files with the analyzing program recommended by ISO 12836. With that, the measurement errors were reduced and the true values of 3D-printed models were focused on. 

For evaluating RMS and inTOL values between DLP, MJP, and SLA techniques, statistically significant differences in trueness were found. Emir et al. reported that the RMS on the accuracy of complete arch measurements showed significant differences between 3D-printed models: the DLP technique (46.2 µm) showed the most accurate results, followed by SLA (51.6 µm) and MJP (58.6 µm) [23]. On the other hand, Kim et al. reported that the accuracy of full arch models fabricated by the MJP technique (62–106 µm) exhibited the highest accuracy, followed by DLP (76–143 µm) and SLA groups (86–141 µm) [24]. This is consistent with the results from this study, where mean MJP (76 ± 20 µm) printing technology exhibited excellent trueness in 4 measured positions, followed by the mean values of DLP (83 ± 26 µm) and SLA (92 ± 21 µm) printers. It was assumed that Kim’s results were also further supported by the measurements of surface roughness from this study. The mean surface roughness in models fabricated by the MJP technique showed the smoothest surface, followed by DLP and SLA. 

Although there were significant differences in the RMS of accuracy (trueness), all tested models in this study showed clinically acceptable values based on the standards of previous studies. The previous studies reported that a dimensional difference less than 500 µm in dental models did not affect clinical decision [25], and for orthodontic and diagnostic purposes, less than 300 µm values are known to be clinically acceptable [26]. However, for the fit of the fabrication of prosthesis, when a printed model shows this much discrepancy, it might not be sufficient because other manufacturing processes of prostheses would increase errors, and the fit of prosthesis is one of most important factors for prognosis of treatment. In the same context, Rossini et al. reported that recommended trueness of digital models for clinical setting should be under 200 µm [27], which is twice the results of this study. Consequently, trueness of 3D-printed models by DLP, MJP, and SLA in the present study showed that all casts could be used for fabrication of prosthesis.

All tested models printed by DLP (244 ± 117 µm), MJP (272 ± 103 µm), and SLA (249 ± 69 µm) techniques showed excellent precision according to superimposition data, as well as ICC values above 0.95. According to the study of Kim et al., MJP and DLP techniques were more precise than the SLA techniques [24]. Even though results from this study showed DLP and SLA techniques were more precise than MJP, no statistically significant differences between groups were found (*p* = 0.381). Clinicians can expect that appropriate models for clinical use are printed, regardless of printing order or number under the same conditions.

Deviations of two points in prepped molar, four points of prepped premolar, and one point of intimate tooth contact were also analyzed to assess the deviations of marginal and contact areas. Marginal fit is the most important feature of fixed prostheses, and marginal misfit can lead to secondary caries, pulpitis as well as periodontal problems including gingivitis and bone loss, causing failure of the prosthesis. Therefore, after producing the prostheses using transmitted STL files, they needed to be checked with the working model to reduce errors before the delivery to patients.

The clinically acceptable marginal fit of fixed prostheses has been reported as 90 μm to 200 μm [16,28] and many researchers consider the optimal marginal fit should be within 120 μm [16]. For proximal contact area, 50 µm contact thickness is typically regarded as appropriate [29]. In addition to that, previous studies demonstrated that the linear deviation of printed model regarded acceptable at 200 µm because the measurement error of the plaster model itself is close to this range [22]. Therefore, if the deviation values of 3D-printed models from this study show less than these values at measured points (margins and a proximal contact), clinical use of 3D-printed models for manufacturing prostheses could be considered clinically acceptable.

In the present study, the mean deviation of margins on molars and premolars were all observed within the acceptable range of previous studies (less than 120 µm), and the MJP group showed the fewest deviations (Table 4). For proximal contacts, all deviation values were also less than 50 µm. Therefore, to check the accuracy of fabricated prostheses with the 3D-printed working model, printing by 3D techniques is recommended regardless of the printing methods. Furthermore, Jeong et al. showed 3D-printed models fabricated by SLA techniques (52 µm) were observed to be more accurate than milled models (152 µm) [30]. In other words, to reduce errors of fabrication of prostheses, reproducing digital models by 3D printing, instead of milling, is a good option regardless of the printing materials. 

The color map showed contraction (blue) and expansion (orange-red) of 3D-printed models in this study. Prepped tooth (molar or premolar) showed contraction in buccolingual and mesiodistal planes regardless of the 3D techniques (Figure 4). Park et al. suggested that the DLP cast tended to contract, whereas casts in the MJP and SLA groups expanded buccolingually [31]. However, in our study, all casts contracted buccolingually. The differences of those results might be because of the differences in geometry of the measured casts; Park et al. measured deviations on a thin cylinder form of a printed model, while prepped casts for three-unit Br were used in this study, which needs more resin materials (volume). This means more shrinkage could have occurred under polymerization. Similarly, absolute deviation values showed that the posterior region (i.e., molar) deviated more than the anterior region (i.e., premolar) (Table 4). This result can be explained by the presence of a higher density of polymers in the posterior region than in the anterior region; more polymer chains in the resin printing and polymerization process might introduce more deviation [32]. These results might correspond with complaints of dental technicians that adaptation of prostheses to 3D-printed working models is often loose.

The layer thickness setting could be the most decisive factor for accuracy of resin printed models, under the condition in which each printer has a determined x and y resolution [33]. For the stacking layer thickness of 3D resin printing, there was a previous study where part of the printed area deviated from the ideal boundary in each layer, and the chances of potential errors increased with the number of additive layers [34]. In other words, the thinnest layer would not be optimal. Zhang et al. reported that the optimal layer thickness of the DLP technique was 50 μm, showing the best balance between surface accuracy and printing error, while for the SLA technique, optimal layer thickness was 25–50 µm [35]. Accordingly, for most accurate results of different printing techniques, this study used a 50-μm stacking layer in DLP and SLA. However, a 32-µm layer was used for MJP, as recommended by the manufacturer. 

A different study concluded that a thinner layer thickness resulted in an increased number of layers, and thus a higher resolution of the z axis [35]. That might explain why MJP (z axis: 790 dpi) showed higher z axis resolution than SLA (z axis: 500 dpi) material used in this study. However, according to the study of Braian et al., high resolution is not equivalent to accuracy [36]. Printers that have high resolution can fabricate models with finer detail; however, other various factors such as layer thickness, number of layers, degree of polymerization shrinkage, polymerizing laser speed and intensity, building direction and angle, thermal changes (expansion or contraction), reference model geometries, supporter design, and post processing can affect the accuracy (trueness and precision) of printed objects on top of the printed materials with different resolutions [22,37,38,39,40,41,42]. In the present study, the overall resolution of SLA (4000 × 4000 × 500 dpi) was reported to be the highest among DLP (1920 × 1080 dpi), MJP (1600 × 900 × 790 dpi), and SLA, according to a manufacturer. However, MJP showed the smoothest surface (Table 8) and significantly higher accuracy than others (Table 2 and Table 5). The building angle, reference model geometries, and supporter design of this study were applied equivalently in DLP, MJP, SLA techniques. Therefore, it was assumed that the degree of polymerization shrinkage of each printing material, as well as the thermal changes of models at printing and post-processing, affected the results of this study. To tackle these effects, further studies are needed.

The cost and printing speeds of devices with different 3D printing technologies vary. In the present study, high-end 3D printers from well-known brands and commercially used 3D printing materials were selected. Even though manufacturing speed was found to be the fastest with the SLA technique in this study, an SLA 3D printer is known to be much more expensive than a DLP printer. Therefore, there need to be consideration about which 3D printing technique is the most cost-effective option to fabricate working digital models with appropriate accuracy. The printing accuracy can also be improved by optimizing the parameter settings. Further studies are needed to reduce deviations and optimize the parameters in various 3D printers.

## 5. Conclusions

Under the limits of this study, MJP casts showed significantly higher accuracy (trueness) than DLP and SLA casts, and there were no significant differences found in precision. Although all casts contracted buccolingually, the overall 3D accuracy of all the 3D-printed resin models produced by the DLP, MJP, and SLA techniques, showed clinically acceptable ranges. The overall deviation values of printed models at 2D planes also showed clinically acceptable ranges; less than 120 µm for margins and 50 µm for contact areas. It was concluded that dental working models printed by DLP, MJP, and SLA techniques can be used for adjusting and determining the fit of fixed prostheses before delivery.

## Figures and Tables

**Table 1:** Specifications of the 3D printer and materials used in this study.

3D Printer Model	Technology	Resin Material	Manufacturer of Printers and Resin Materials	Stacking Layer Thickness (µm)	Time	Building Angle (Degree)
Nextdent 5100	DLP	Next Dent model 2.0	3D systems	50	3 h 40 m	0
ProJetMJP 2500+	MJP	Visijet M2R-WT (SL7810)/Visilet M2 Sup	3D systems	32	3 h 42 m	0
ProJetSLA 7000	SLA	Accur ABS white (SL7810)	3D systems	50	43 m	0

**Table 2:** The accuracy (trueness by RMS value (mean ± SD) mm) of 3D-printed models (*n* = 36).

Positions	DLP (mm)	MJP (mm)	SLA (mm)	*p*-Value
#27 buccolingul	0.117 ± 0.007 ^a^	0.096 ± 0.029 ^b^	0.101 ± 0.004 ^b^	<0.001
#25 buccolngual	0.064 ± 0.009 ^c^	0.054 ± 0.002 ^d^	0.066 ± 0.005 ^c^	<0.001
Proximal contact	0.052 ± 0.011 ^e^	0.059 ± 0.012 ^e^	0.078 ± 0.021 ^f^	<0.001
#27–25 mesiodistal	0.100 ± 0.022 ^h^	0.098 ± 0.010 ^h^	0.123 ± 0.008 ^g^	<0.001

Different letters indicate statistically significant difference based on Mann-Whitney test at *p* < 0.017.

**Table 3:** The inTOL (%) of trueness (mean ± SD) for 3D-printed models (*n* = 36).

Positions	DLP (%)	MJP (%)	SLA (%)	*p*-Value
#27 buccolingual	81.2405 ± 6.623 ^a^	84.917 ± 2.874 ^b^	84.335 ± 2.501 ^b^	0.037
#25 buccolingual	88.776 ± 8.189 ^c^	93.661 ± 1.832 ^d^	88.227 ± 2.537 ^c^	0.01
Proximal contact	95.488 ± 2.447 ^e^	92.371 ± 4.106 ^f^	84.050 ± 5.198 ^g^	<0.001
#27–25 mesiodistal	85.292 ± 2.799 ^h^	88.705 ± 0.790 ^i^	83.086 ± 2.775 ^j^	<0.001

Different letters indicate statistically significant difference based on Mann-Whitney test at *p* < 0.017.

**Table 4:** The absolute values of deviations for 3D-printed models at margins and contact area.

Positions	DLP (mm)	MJP (mm)	SLA (mm)	*p*-Value
molar (buccolingual)	0.100 ± 0.022 ^a^	0.0291 ± 0.020 ^b^	0.070 ± 0.015 ^c^	<0.001
premolar (buccolingual)	0.074 ± 0.033	0.068 ± 0.029	0.074 ± 0.044	0.625
molar (mesiodistal)	0.109 ± 0.015 ^d^	0.020 ± 0.012 ^e^	0.051 ± 0.044 ^f^	<0.001
premolar (mesiodistal)	0.029 ± 0.028	0.029 ± 0.014	0.029 ± 0.014	0.996
contact to proximal tooth	0.036 ± 0.015 ^g^	0.009 ± 0.009 ^h^	0.036 ± 0.015 ^g^	<0.001

Different letters indicate statistically significant difference based on ANOVA at *p* < 0.05.

**Table 5:** The accuracy (precision by RMS value (mean ± SD) mm) of 3D-printed models (*n* = 30).

DLP (mm)	MJP (mm)	SLA (mm)	*p*-Value
0.244 ± 0.117	0.272 ± 0.103	0.249 ± 0.069	0.381

**Table 6:** The inTOL (%) of precision (mean ± SD) for 3D-printed models (*n* = 30).

DLP (%)	MJP (%)	SLA (%)	*p*-Value
95.596 ± 2.345	95.134 ± 1.726	95.879 ± 0.988	0.643

**Table 7:** Intraclass Correlation Coefficient (ICC) values of 3D-printed models.

DLP	MJP	SLA
0.996	0.955	0.992

**Table 8:** Mean surface roughness ((mean ± SD) µm) on surface of 3D-printed models.

Measurement	DLP (µm)	MJP (µm)	SLA (µm)
Ra	4.123 ± 2.531	2.345 ± 0.374	7.75 ± 2.478
Sa	56.75 ± 21.132	31.25 ± 14.07	77.3 ± 7.912

## Data Availability

Data sharing is not applicable.
